# Foreign Summary

**Published:** 1840-01-04

**Authors:** 


### FOREIGN SUMMARY.

### *Clinical Remarks, by* Sir Benjamin C. Brodie, *at St. George's Hospital.*

Ulcers.—I shall make but a few general observations upon ulcers, as I shall have to speak to you at large upon some of the particular species of these morbid degenerations of structure. It is somewhat difficult to give a correct definition of what an ulcer really is. We say that a part is ulcerated, when some portion of the solid structure is absorbed, and the exposed surface presents a suppurating aspect. Internal ulcers, as they are sometimes termed, come more particularly under the care of the physician; but, as I have just said, ulcers on the external surface of the body, not depending on any poison as a cause of their presence, are very difficult to describe in words, on account of the great variety that are presented to the observation of the surgeon. I will first, then, speak of

*Healthy Ulcers.*—These are such as may be caused by the application of caustic, or from an incision made with a knife. These ulcers secrete a thick white pus, of the colour of cream, the granulations are small and pointed, and of a florid red colour. Generally speaking, an healthy ulcer will heal of its own accord; but it is generally necessary to apply some simple dressing to it, and place a roller over this, as it will prevent its scabbing. When the ulcer is of a large size, you will heal it best by the application of stimulants. These substances vary, but, they have all one common purpose. They are generally used in the form of diluted nitric acid, or a solution of sulphate of copper, or sulphate of alum; but, perhaps, none are so efficacious as a solution of nitrate of silver, in the proportion of two grains to one ounce of water. There is very great discretion necessary in the application of these stimulants. The ulcer may be over-stimulated, or the reverse may be the case. When the ulcer is of a large size, its cicatrization may be promoted by gently purging the patient, and applying straps of adhesive plaster to the ulcer. An ulcer in the neck, caused by a burn, will heal; but if great care is not taken, the cicatrix will contract, and the chin will be bound down to the sternum. This, of course, causes great inconvenience to the patient, and you may perhaps imagine that the relief is very easy, and say, "cut across the cicatrix,"—ay, ay, you may cut across the cicatrix if you like, but it will form again, and become still more contracted. It was in such cases as these that the late Mr. Earle proposed to dissect out the cicatrix, and bring the parts together in such a manner that the contraction shall not be productive of any inconvenience. I have performed this operation of excising the cicatrix in several instances; but I cannot say that I have ever been perfectly satisfied with the result. There are some appearances which ensue after burns, and which very much resemble ulcers, but they are not ulcers in reality; these are best treated by sprinkling a little powdered prepared calamine on them. There are some ulcers which do not heal very readily; this generally results from weakness of parts. In such cases the granulations are large, and grow very rapidly; they are pale in appearance, and possess but little vascularity, whilst their surface is soft, spongy, and very irregular. Now, in such a case as this, you must restore the strength of your patient's constitution, by giving him bark and such other tonics as he requires. You will do much in this way, but you will do more by attending to the local treatment of the ulcer. If you find the granulations grow above the surface of the skin, you must touch them with the solution of the nitrate of silver, or with the oxidized ointment of nitrate of mercury, and strap it with adhesive plaster. If you find an ulcer become very irritable, you may make pressure on it with adhesive plaster and a bandage; this is an old, but a very excellent way of treating these cases. The adhesive plaster should be spread on cloth, and cut into strips an inch in breadth. Apply one strip some distance below the wound, the next half way over the first, the next half way over the second, and so on, till you have arrived over the ulcer. Take care that the pressure is equal; much pressure is not needed in these cases. When there is much discharge, the adhesive plaster should be changed daily; when there is not much, every other day will be quite sufficient.

*Indolent Ulcers.*—You will meet with these most frequently in the leg. They generally occur a little below the level surface of the skin, have no granulating surface, and the discharge from them is not of pus, but of flakes of coagulable lymph. The edges of the surrounding skin are thick, prominent, and smooth, on their upper surface. If such an ulcer as this comes under your care, put your patient to bed, apply fomentations and poultices, and when the inflammation has subsided, apply some adhesive plaster to the ulcer, with a roller over it. Or you may, if you please, use some stimulating ointment to the part.

*Sloughing Ulcers.*—These are ulcers that spread partly by suppuration, and partly by absorption. They are attended with considerable pain and surrounding inflammation, and the discharge from the ulcerated surface is very copious and offensive. These ulcers cause great constitutional disturbance. The pulse becomes quick, the skin is hot and dry, and the tongue furred. In treating such an ulcer, you must first ascertain what the constitutional cause may be, whether it may arise from the internal irritation of mercury or any other poison in the system. The patient should be kept in a perfect state of quietude. When there is much pain, and a line of limitation between the sloughing and healthy surface shows itself, you will find that the internal use of opium in the dose of from four to five minims of the tincture will prove of great service. There are some of these cases which are benefitted by bark. When the ulcer is not very painful, and the pulse is feeble, you will find the tonic plan of treatment to be the best. When there is a great deal of sloughing, and the skin is dry and hot, you will aggravate the disease by giving stimulants. You must in such a case take blood from the arm, which you will find has the buffy coat; with this you must combine purgatives and diaphoretics. The local treatment may consist of cold applications or the white way ointment, or the chlorate of soda; but I would particularly recommend to you the compound tincture of benzoin. To apply this, dip a piece of lint in the tincture and place it over the ulcer, and prevent it from evaporating, by putting dry lint over it. When the sloughing has stopped, merely wash the parts with the tincture, and apply adhesive strapping over it. There is no general rule of treatment, for according to the external appearance of the ulcer, and to the constitutional disturbance of the system so must be the remedies. Another mode of cure is to stop the sloughing of the ulcer, by applying the concentrated nitric acid to it; but I prefer the mode of cure by the compound tincture of benzoin. In France they use the actual cautery to these ulcers.

*Irritable Ulcers.*—These generally begin in the form of an eruption, terminating in an ulcer which is extremely painful, and sometimes bleeds, and has jagged edges. The chief characteristics of this species are, extreme pain and a great indisposition to heal, and their depending upon a cachectic diathesis of system. Different cases require different treatment. The most general mode of treating these ulcers, is in exhibiting small doses of mercury with the decoction of sarsaparilla. Sometimes the digestive organs are affected, and then you must give bitters. You must bear in mind that these ulcers always depend upon a cachectic state of constitution. The local application may consist of a carrot poultice, with one drachm of the extract of hemlock beat up with it. In some cases Peruvian bark may be applied in powder to the ulcer. Stimulating ointments are sometimes beneficial, whilst at other times they are injurious. The difference between sloughing ulcers and irritable ulcers is this, that the former affect the constitution, and cause the disturbance, whilst the latter are caused by the cachectic state of the constitution.

*Mortification.*—Inflammation may terminate in mortification; but mortification may arise from a number of other causes, as well as inflammation. Some particular inflammations are more likely to terminate in mortification than others, as when they are produced by the bites of venomous reptiles or from wounds. A local injury may bring on inflammation, and this may bring on mortification; but an injury may be so great that the part which receives the injury loses its vitality at once, therefore local injury may bring on mortification in two ways; first, by producing inflammation, and then the inflammation terminating in mortification; and secondly, by destroying the vitality of the part at once. Some parts of the body are more liable to become mortified than others. These parts are the cellular membrane, because it has a lower vitality than some other parts; therefore it is more disposed to become mortified than the skin, because it has less vitality. The skin more readily mortifies than the muscles, for the same reason; this is often proved from the effect of blows on, or fractures of the leg and thigh. The integuments swell up, and after a day or two, you will feel an emphysematous crackling beneath the skin when you press upon it. Now, in such a case, if you cut down upon the cellular membrane beneath the skin, you will find it dead. Not only are some parts of the body more disposed to mortify than others, but there are some constitutions which are more predisposed to take on mortification than others. Those who are addicted to drinking ardent spirits; those whose constitution has been shattered by repeated attacks of disease, or indulgences in vicious habits, are more prone to mortification after the receipt of a local injury, so much so, that in all such cases you will find that the effect resulting from the injury is beyond all proportion to its cause. If any fluid, as urine for instance, gets into the cavity of the peritoneum, it brings on inflammation; but if it gets into the cellular membrane, you will find that mortification will come on; you will find the patient's pulse will be full and frequent, and not hard, as you might expect in inflammation, and that the part is swollen. You put your hand over it, and you feel, as I have before said, an emphysematous crackling upon making slight pressure; the skin is hot, and if the inflammation be extensive, there is hiccough, the belly is blown up with air, the pulse soon gets feeble, delirium ensues, and the patient dies. Where there is internal mortification, the symptoms are the same. If the patient dies of mortification of an external part, it is generally the cellular membrane that is mortified. But will not the skin also get mortified? Most certainly it will, if you do not prevent it. And how do you prevent it? Why, by making free scarifications through the skin down to the cellular membrane, and setting free the tension under which it labours, and if you do this in good time, you will generally prevent the progress of the mortification altogether.

Whenever you suspect that the cellular membrane is mortified (and you will have the emphysematous crackling when that occurs,) make free scarifications, and let out the putrid exhalations beneath, which are poisoning the system. Having made the scarifications, the other local treatment should consist of poultices and fomentations. You may apply as a wash a solution of the chloride of soda; if it does no other good, it will take off the offensive putrid odour of the parts.

With respect to the constitutional treatment, no general rule can be laid down. In some cases, where there is much inflammatory action, purgatives and diaphoretics must be given; but when the inflammation has subsided, you must leave off the anti-phlogistic remedies, and give stimulants, to excite the system, but never to such an extent as to produce fever. For such a purpose you may administer either opium or ammonia. But suppose that a mortified part separates, we call that the process of sloughing. We know but very little of the physiology of this process, which may be described best to you as a peculiar form of ulceration. Whilst this process is going on, you must encourage it by the constant application of fomentations and poultices. Ulcers may perhaps form from the scarifications you have made, and these must be treated according to the rules I laid down to you when speaking of them. But you will, perhaps, ask—may you not amputate when mortification is going on? I think you may. Of course you would not amputate when mortification in the cellular membrane is complete; you are told to scarify, and what, I would ask, is amputation, but scarification in a more extended sense of the word? When mortification is the consequence of contusion, it is to be treated as contusion. You will feel the emphysematous crackling of mortification; scarify the parts therefore immediately, and you will save the skin.

Old persons are very subject to ossification of the arteries; the femoral, popliteal, and peroneal arteries sometimes ossify; and if the ossified arteries contract, the patient will be very likely to have inflammation of the toes, terminating in mortification. The symptoms by which you may know this are pains in the toes; the foot and leg will become œdematous; or perhaps, from a trifling accident, some inflammation is produced, and in both these cases mortification comes on; at another time, perhaps, the patient finds his feet get very cold; he immediately applies external warmth, and takes some internal stimulant, and the mortification is averted; but, if on the other hand, it be neglected, the foot becomes white and cold, the patient complains of great pain, and the toes begin to mortify at their extremities; in either way the progress of the mortification is different. It is sometimes rapid, when it obtains the title of "acute," the skin will become hot, the tongue loaded, the pulse rapid and frequent, the mortification will spread over the foot, delirium will ensue, and death will be the result; or it may, on the other hand, be slower in its progress, when you will have a feeble pulse and a cold clammy skin; the patient falls into a state of coma, and dies. The mortification that comes on in the toes, from ossification of the arteries in the leg and thigh above, may be very likely to destroy life, but will not always do so. When, in such a case, inflammation ends in mortification, if the pulse indicates fulness, it may become a question whether blood should be taken from the arm. The result of my experience in such cases is, that such a practice would prove unsuccessful to the safety of the patient. With respect to local remedies in such a case, you must keep the parts warm; if there be, however, much heat and inflammation about them, you must apply cold lotions, or warm fomentations and poultices, according to circumstances. Some surgeons recommend you to rub the leg above with stimulating liniments: but I do not think that they do any good. Keep your patient quiet in bed; if there be much pain, give him *opium;* I do not know that it is of much service, except where there is severe pain, when you must give it in large doses, in the proportion of one grain every four hours. You may give stimulants internally, for although you cannot increase the diameter of the ossified arteries, you can increase the velocity of the circulating blood. For this same purpose you may give your patient a little wine, but not sufficient, of course, to produce fever. In cases of this kind, where persons have been accustomed to drink a great deal of wine, it may be necessary sometimes to give them as much as a bottle a day. My own opinion as to the employment of stimulants in these cases, induces me to prefer ammonia to all others. You may, for this purpose, give your patient six grains of carbonate of ammonia every four hours. The parts affected with mortification become putrid, and in process of time they separate. This process of separation occurring in the foot, is very slow and tedious, because it has to slough through tendons, ligaments, and bones. It therefore becomes a question, whether you are to amputate in these cases of mortification, caused by ossified arteries, and when are you to do it? If things proceed favourably, the parts will separate in time of their own accord, and nature will make a very good stump, with a little assistance of the surgeon's saw in getting through the bone. But there is another question, with reference to this subject, and that is, should you amputate during the progress of mortification? I always oppose it. I think that you should never amputate under these circumstances; but it is done, however, in some cases by some surgeons. In old persons who die of mortification of the toes, the femoral artery is very commonly contracted in its diameter. Now, whether this is caused by inflammation of its coats or not, I do not know.

There is another disease of the arteries causing mortification, but I have only seen three cases of the kind. One was an obliteration of the artery, caused by inflammation of its coats; the symptoms were pain and tenderness in the course of the artery. In this case the sloughing process proceeded favourably, and the parts separated down to the bone which I sawed through, and a good stump was formed. Now, why does not mortification ensue after applying a ligature to the femoral artery? If you tie an artery, the obliteration is only in the trunk of the artery, but not in the anastomosing branches; but in a case like the one I have just named, it is not only in the main trunk, but in the branches of that main trunk also. Mortification will also occur in the toes after typhus fever. In such a case, it is, of course, owing to a languid circulation, and from this cause parts at the extremities may also become mortified, after other severe diseases. Besides the causes which I have already mentioned, there are various others that may produce mortification, such as the actual cautery, and the various caustics, which act by destroying the vitality of the part to which they are applied. The actual cautery acts mechanically, and the caustics chemically; but there is one caustic, however, which operates only on the vitality of the part to which it is applied, and not chemically: this is arsenic. The other caustics act on the dead body, but arsenic does not. Mortification, when caused by the use or abuse of these caustics, requires the same local treatment which I have before described; you must apply poultices and fomentations, and wait for the process of suppuration. Indeed, in such cases as these, a surgeon should do but little. He should look to the constitution, and judge if it is able to go through what is required; if so, you should let nature effect the cure, but if the constitution be too weak to bear the process of the separation of the parts, then the surgeon must act according to the circumstances which present themselves. Of the surgical nomenclature used for this affection, I should have spoken to you first. You will meet in authors with the words gangrene and sphacelus. The first of these signifies that period when the parts are not quite dead; the second means that period when the mortification is complete. From gangrene the patient may recover; from sphacelus never.—*London Lancet November* 9.

---

*National Medical Convention.*—The third decennial National Medical Convention for the revision of the Pharmacopœia of the United States, assembled in the City Hall, Washington, on the 1st January, 1840.

The following Medical Societies and Colleges were represented in the Convention, viz.: The Rhode Island Medical Society; the New Jersey Medical Society; the College of Physicians of Philadelphia; the University of Pennsylvania; the Jefferson Medical College; the Delaware Medical Society; the Washington University of Baltimore; the Medical and Chirurgical Faculty of Maryland; the Medical Society of the District of Columbia; the Columbian Medical College; the Vincennes Medical Society of Indiana, and the Georgia Medical Society. The credentials of the delegations from the White Mountains' Medical Society of Vermont, from the Medical Society of New Hampshire, from the Albany Medical Society, and from the College of Physicians and Surgeons of Lexington, Kentucky, were presented by the President, but the Delegates were prevented from attending.

The Convention elected Lewis Condict, M. D., of New Jersey, President; George B. Wood, M. D., of Philadelphia, Vice President; N. W. Worthington, M. D., of Georgetown, D. C., Secretary; Harvey Lindsly, M. D., of Washington city, Assistant Secretary.

The chief object of the Convention being the revision and emendation of the Pharmacopœia of 1830, this subject engaged its attention primarily, and, after mature deliberation and a free interchange of opinion among the delegates from different parts of the Union, the Convention referred to a committee of seven members all the documents in its possession, with instructions carefully to revise, prepare, and publish the Pharmacopœia of the United States for 1840, under the authority of this Convention.

Arrangements were also made for the assembling of the fourth decennial Convention in Washington, on the first Monday in May, 1850.

Having passed votes of thanks to the officers of the Convention, and to the Board of Aldermen for the use of their room, the Convention, after a session of three days, adjourned.—*Nat. Int.*

